# Parkinson’s disease laterality: a ^11^C-PE2I PET imaging study

**DOI:** 10.1007/s00415-020-10204-y

**Published:** 2020-09-02

**Authors:** Andreas-Antonios Roussakis, Zhou Zeng, Nicholas P. Lao-Kaim, Antonio Martin-Bastida, Paola Piccini, TRANSEURO consortium, Roger A. Barker, Krista Farrell, Natalie Valle Guzman, Xiaoling He, Stanley E. Lazic, Sarah Moore, Robert Morris, Pamela Tyers, Ruwani Wijeyekoon, Danielle Daft, Sam Hewitt, Biswas Dayal, Thomas Foltynie, Zenobia Kefalopoulou, Philipp Mahlknecht, Hjalmar Bjartmarz, Anders Björklund, Olle Lindvall, Jenny Nelander-Wahlestedt, Malin Parmar, Gesine Paul, Hakan Widner, Alistair Church, Stephen Dunnett, Kathryn Peall, Anne Rosser, Jean Marc Gurruchaga, Stéphane Palfi, Tobias Piroth, Christian Winkler

**Affiliations:** 1 Division of Neurology, Neurology Imaging Unit, Hammersmith Hospital, Imperial College London, Du Cane Road, London, W12 0NN UK; 2 Second Xiangya Hospital of Central South University, Changsha, Hunan People’s Republic of China; 3 Department of Neurology and Neurosciences, Clinica Universidad de Navarra, Pamplona, Madrid, Spain

**Keywords:** Parkinson’s disease, Asymmetry, Positron emission tomography, Striatum, Dopamine transporter

## Abstract

Asymmetry of striatal dopaminergic deficits and motor symptoms is a typical characteristic of idiopathic Parkinson’s disease (PD). This study aims to characterise the trend of asymmetry in moderate-stage PD. We performed a 19-month longitudinal study in 27 patients with PET-CT imaging and appropriate clinical assessments. ^11^C-PE2I non-displaceable binding potential (BP_ND_) was calculated bilaterally for the striatum at baseline and follow-up to estimate the in vivo density of striatal dopamine transporters (DAT). Changes in striatal ^11^C-PE2I BP_ND_ over time were more prominent in the ipsilateral as compared to contralateral side. Changes in MDS-UPDRS-III (motor component of the Movement Disorders Society Unified PD Rating Scale) were not different between the clinically most and least affected body sides. Our data support that the asymmetry in striatal dopaminergic degeneration becomes less prominent in moderate-stage PD. In contrast, during the above period, the asymmetry of motor symptoms was maintained between the clinically most and least affected body sides.

## Introduction

Asymmetry of striatal dopaminergic deficits and subsequent lateralisation of motor symptoms are key features of idiopathic Parkinson's disease (PD) [1] and as such are considered supporting factors during the diagnostic process [2]. Remarkably, after decades of research, the underlying causes of PD asymmetry remain poorly understood. Some consider it coincidental, while others have drawn links to handedness, certain genetic loci and environmental factors [3–6].

Asymmetry of motor symptoms has been shown to correspond to asymmetry of striatal dopaminergic deficits in early PD [7, 8]. PET studies show that the degree of asymmetry in striatal dopamine transporter (DAT) tracer binding becomes less prominent over the course of the disease [7, 9]. However, clinical studies indicate that the motor symptoms retain asymmetry over many years [10, 11].

To our knowledge, there are currently no longitudinal studies evaluating the progression of asymmetry in clinical symptoms and dopaminergic integrity in moderate PD. To address this, we performed serial clinical ratings and dopaminergic brain imaging over a 19-month period. To evaluate dopaminergic terminal integrity, we use ^11^C-PE2I [^11^C *N*-(3-iodopro-2E-enyl)-2β-carbomethoxy-3β-(4ʹ-methylphenyl) nortropane] PET, which has been shown to have high specificity for the dopamine transporter (DAT) [12]. To monitor clinical progression, we use the Movement Disorders Society Unified PD Rating Scale (MDS-UPDRS) [13].

Based on previous reports [7, 10, 11, 14], we hypothesise that in moderate PD, the decline of striatal DAT density in the ipsilateral side is greater than in the contralateral side, and that the asymmetry of striatal DAT degeneration becomes less marked over 19 months. We expect the progress of clinical scores to occur in a similar pattern between the clinically most and least affected body sides.

## Methods

### Participants

Patients with idiopathic PD were recruited from specialist movement disorders clinics. All patients fulfilled the Queen Square Brain Bank diagnostic criteria for idiopathic PD [15], did not have a history of depression and/or cognitive impairment (Mini-Mental State Examination scores < 26) and were not being treated with medications acting directly on the serotonergic system or dopamine transporters. Levodopa equivalent doses (LED) were calculated using conversion factors reported in a previous study [16]. The OFF medication state was defined by the withdrawal of dopaminergic medication for at least 24 h for standard release and 48 h for prolonged-release preparations.

### Clinical characteristics

Motor severity was evaluated using the motor component of the MDS-UPDRS scale (MDS-UPDRS-III) [13] and staging was assessed with the modified Hoehn & Yahr (H&Y) rating scale [17]. The same clinical team performed baseline and follow-up clinical assessments whilst participants were in the OFF medication state. To identify the clinically most and least affected body sides at enrolment, lateralization scores were calculated separately based on bilateral components of the MDS-UPDRS-III (rigidity, finger tapping, hand movements, pronation-supination movements of hands, toe tapping, leg agility, postural or kinetic tremor of the hands, rest tremor amplitude; maximum lateralized score = 44) [18].

All patients provided their written consent. This study was carried out in accordance with the Declaration of Helsinki and was approved by the Health Research Authority, the NRES Research Ethics Committees of the UK (REC 12/EE/0096 and 10/H0805/73), and the UK Administration of Radioactive Substances Advisory Committee.

### ^11^C-PE2I PET-CT and MRI acquisition and pre-processing

All scans were conducted at Invicro Ltd Research Imaging Facility in London, UK. Patients underwent ^11^C-PE2I PET-CT and MR imaging twice: at baseline and follow-up (after 19 months) in the OFF medication state.

MRI scans were acquired on a 3T Siemens Magnetom Trio system with 32-channel head coil. T1-weighted magnetization-prepared rapid acquisition gradient-echo (MPRAGE) images were obtained (repetition time = 2300 ms, time echo = 2.98 ms, flip angle of 9°, time to inversion = 900 ms, slice thickness, 1 mm; matrix size, 240 × 256 mm) for co-registration with the PET images. One whole-brain volume was acquired consisting of 160 slices lasting 301 s.

^11^C-PE2I PET scans were obtained on a Siemens Biograph TruePoint HI-REZ 6 PET/CT system (Siemens Healthcare). A low-dose CT transmission scan (0.36 mSv) was performed for attenuation-correction purposes. ^11^C-PE2I (mean radioactivity dose of 319.25 ± 38.77 MBq) was intravenously administered as a bolus injection and dynamic emission data were acquired for 90 min after injection. 26 temporal frames were reconstructed using a filtered back-projection algorithm (direct inversion Fourier transform; matrix size: 128 × 128, zoom: 2.6, 5 mm transaxial Gaussian filter, pixel size: 2 mm isotropic). All PET imaging analyses were carried out using Molecular Imaging and Kinetic Analysis Toolbox software package for academic use (MIAKAT™: www.miakat.org), implemented in MATLAB (Mathworks, Natick, MA) and utilising FSL 6.0 (FMRIB Image Analysis Group, Oxford, UK) [19] and SPM12 (Statistical Parametric Mapping, Wellcome Trust Centre for Neuroimaging, London, UK) functions.

Structural MPRAGE images were segmented and rigid-registered to the standard Montreal Neurological Institute (MNI) template. Registered MPRAGE images were then used to manually trace striatal (putamen and caudate) regions of interest (ROIs) in Analyze 11.0 (Biomedical Imaging Resource, Mayo Clinic). An MNI-based regional atlas (CIC Atlas v1.2; GlaxoSmithKline Clinical Imaging Centre, London, UK) was non-linearly warped to the registered MPRAGE images to define the cerebellum, which was used as the reference region. Rigid-body registered segmentation images were used to enable masking of the gray matter of cerebellum.

Dynamic PET images were motion-corrected by rigid registration to reference frame 16, chosen due to its high signal-to-noise ratio [12]. Signal-averaged (summed) images were then created and co-registered to the corresponding MPRAGE images. The parameters were applied to the realigned dynamic PET images so that all images were in mutual space. Regional time-activity curves were created from the realigned and registered dynamic PET frames. The simplified reference tissue model with the cerebellar grey matter as reference region was used to calculate regional ^11^C-PE2I non-displaceable binding potential (BP_ND_) [20–22]. We calculated ^11^C-PE2I BP_ND_ values for the putamen and caudate bilaterally. The *contralateral* and *ipsilateral* striatal ROIs were defined according to the clinically most affected body side at baseline.

### Statistical analyses

All statistical analyses were performed using SPSS v22.0 (SPSS Statistics for Macintosh; IBM Corp., Armonk, NY). Three-way repeated-measures analysis of covariance (ANCOVA) was conducted with ^11^C-PE2I BP_ND_ as the dependent variable, age and gender as covariates, and three factors: time (baseline, follow-up), side (contralateral, ipsilateral), and region (putamen, caudate). Further two-way repeated ANCOVAs were performed for the caudate and putamen separately, each including time (baseline, follow-up) and side (contralateral, ipsilateral) and co-varying for age and gender. Two-way repeated ANCOVA was also used to examine the effect of time (baseline, follow-up) and side (contralateral, ipsilateral) on MDS-UPDRS-III scores, controlling for age and gender. Bonferroni adjustments were applied where appropriate. The level of statistical significance was set at *α* = 0.05. Normal distribution was evaluated using Shapiro–Wilk test and visually by means of residual QQ plots.

The relationships between lateralised ^11^C-PE2I BP_ND_ values and corresponding MDS-UPDRS-III motor scores were examined using Pearson’s correlation coefficient. Correlations between changes (follow-up—baseline) in lateralised ^11^C-PE2I BP_ND_ values and corresponding changes in MDS-UPDRS-III motor scores were conducted using Spearman’s rho, as several variables demonstrated significant deviation from normality (Shapiro–Wilk, *p* < 0.05) [23]. Comparisons of H&Y staging between baseline and follow-up were performed with Wilcoxon signed-rank test.

## Results

### Participant characteristics

Demographic and clinical characteristics of PD patients are summarised in Table 1. 36 PD patients were recruited at baseline. Of those, 9 participants were lost to follow-up at 19 months, thus leaving 27 evaluable patients for inclusion in this study. As expected, at the end of the follow-up period, all PD patients (*n* = 27) were on significantly higher levodopa equivalent doses (LED) and had higher MDS-UPDRS-III scores (*p* < 0.05). During this period, the H&Y staging remained unchanged (*p* > 0.05).

**Table 1:** Patient demographics and clinical characteristics at baseline and follow-up

	Baseline (*n* = 27) Mean, SD	Follow-up (*n* = 27) Mean, SD	Statistical significance
Age (years)	55.18, 6.82	56.73, 6.73	–
Disease duration (years)	5.53, 1.88	7.08, 1.87	–
LED_TOTAL_ (mg)	694.21, 350.82	837.17, 363.19	*
LED_Ldopa_ (mg)	398.48, 346.76	508.21, 396.86	*
MDS-UPDRS-III	31.59, 10.70	36.37, 10.15	*
Hoehn and Yahr scale	1.96, 0.19	2.04, 0.19	ns
MMSE	29.74, 0.57	29.74, 0.64	ns

*LED* levodopa equivalent doses, *ns* not significant, *MDS-UPDRS* Movement Disorders Society Unified Parkinson's Disease Rating Scale; *MDS-UPDRS-III* motor component of MDS-UPDRS; *MMSE* Mini-Mental State Examination

*Significant at *p* < 0.05; comparison between follow-up and baseline; paired sample *t* tests or Wilcoxon signed-rank test

(Table 1).

### Clinical data analyses

**Fig. 1:**
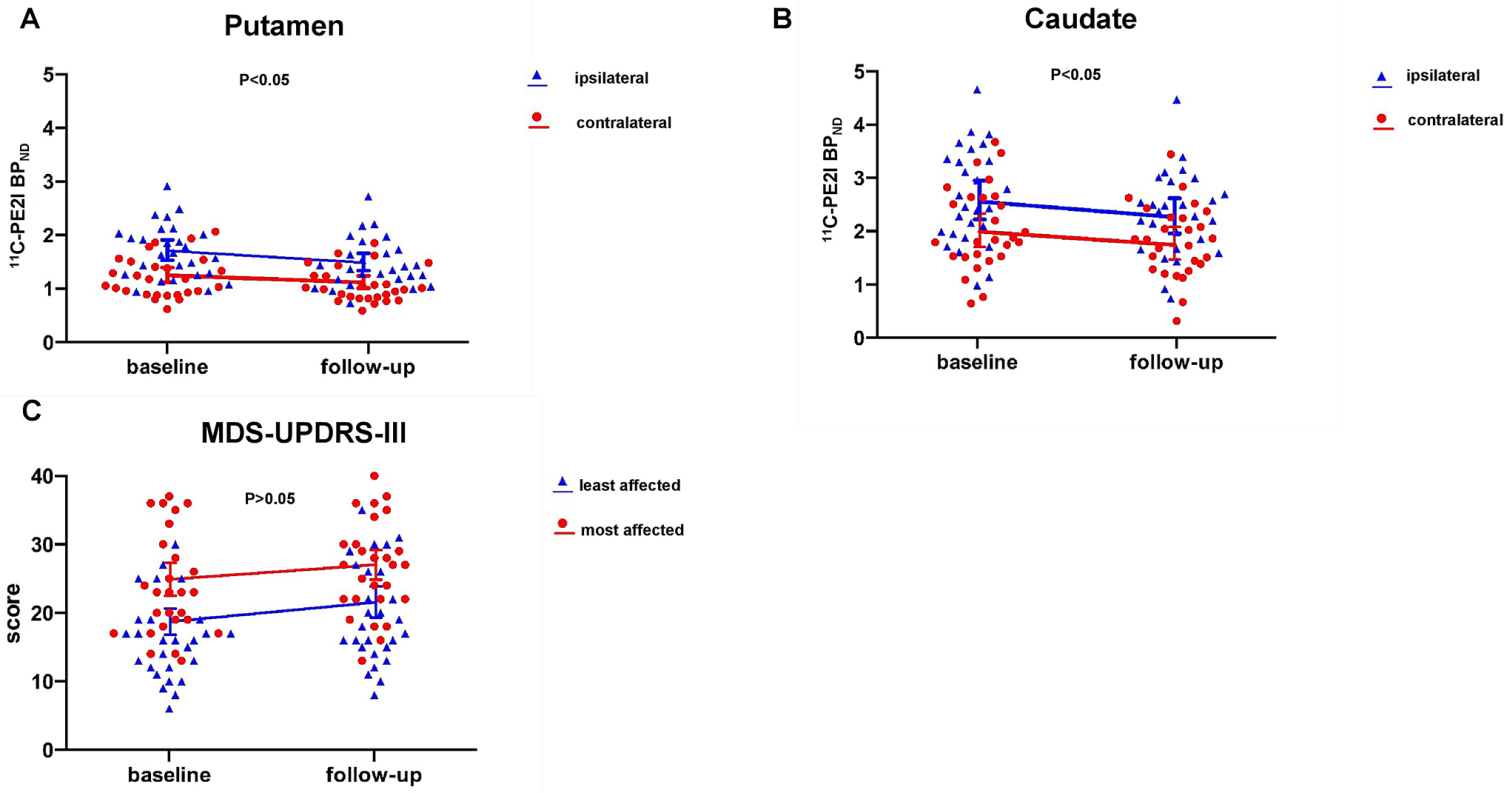
Changes in striatal ^11^C-PE2I BP_ND_ values and motor severity (MDS-UPDRS-III scores) over 19 months. A significant time × side interaction was observed for striatal ^11^C-PE2I BP_ND_ values (*p* < 0.05; repeated measure analysis of covariance; **a**, **b**) but not for MDS-UPDRS-III scores (**c**). Dots represent the individual patients; lines connect baseline and follow-up mean values. *BP*_*ND*_ non-displaceable binding potential, *MDS-UPDRS* Movement Disorders Society Unified Parkinson's Disease Rating Scale, *MDS-UPDRS-III* motor component of MDS-UPDRS, *least affected* the least clinical affected body side, *most affected* the most clinical affected body side

The progression of motor severity (MDS-UPDRS-III scores) over 19 months is shown in Table 2 and Fig. 1. Repeated ANCOVA did not reveal a time × side interaction for MDS-UPDRS-III scores [*F* (1, 24) = 0.54, *p* = 0.47], showing that the changes in MDS-UPDRS-III scores over time were not significantly different between the clinically most and least affected body sides. A significant main effect of side was observed [*F* (1, 24) = 142.78, *p* < 0.001], with higher MDS-UPDRS-III scores found for the clinically most as compared to least affected body sides. A significant main effect of time [*F* (1, 24) = 11.02, *p* < 0.05] indicated that MDS-UPDRS-III scores were higher at follow-up than at baseline (Table 2).

**Table 2:** MDS-UPDRS-III and striatal ^11^C-PE2I BP_ND_ values at baseline and follow-up

	Baseline Mean, SD	Follow-up Mean, SD	Statistical significance
MDS-UPDRS-III (19.31 months ± 4.15)			
Most affected	23.93, 7.37	26.59, 6.76	*
Least affected	16.22, 5.65	19.75, 7.09	**
^11^C-PE2I BP_ND_ (18.93 months ± 3.68)			
Putamen			
Contralateral	1.22, 0.07	1.07, 0.06	***
Ipsilateral	1.71, 0.09	1.47, 0.08	***
Caudate			
Contralateral	2.06, 0.15	1.81, 0.12	**
Ipsilateral	2.65, 0.17	2.33, 0.15	**

*BP*_*ND*_ non-displaceable binding potential, *MDS-UPDRS* Movement Disorders Society Unified Parkinson's Disease Rating Scale, *MDS-UPDRS-III* motor component of MDS-UPDRS, *least affected* the least clinical affected body side, *most affected* the most clinical affected body side

^*^Significant at *p* < 0.05; **significant at *p* < 0.01; ***significant at *p* < 0.001; comparison between the follow-up and baseline; paired sample *t* tests

### Striatal ^11^C-PE2I BP_ND_ analyses

The decline in ^11^C-PE2I BP_ND_ values over 19 months is shown in Table 2 and Fig. 1. Three-way repeated-measures ANCOVA, did not reveal a significant time × side × region interaction for ^11^C-PE2I BP_ND_ values [*F* (1, 24) = 1.10, *p* = 0.31] nor a significant time × region interaction across levels of side (contralateral, ipsilateral) [*F* (1, 24) = 0.14, *p* = 0.71]. However, there was a significant time × side interaction on ^11^C-PE2I BP_ND_ values across levels of region (putamen, caudate) [*F* (1, 24) = 4.38, *p* < 0.05]. A main effect of region [*F* (1, 24) = 89.91, *p* < 0.001] indicated ^11^C-PE2I BP_ND_ values were significantly lower in the putamen as compared to the caudate.

Two-way repeated-measures ANCOVAs were also conducted for the putamen and caudate, separately. For the putamen, a significant time × side interaction [*F* (1, 24) = 6.32, *p* < 0.05] revealed uneven effects of time on ^11^C-PE2I BP_ND_ values between contralateral and ipsilateral sides. Significant simple effects of time (*p* < 0.001) were observed, with significantly lower ^11^C-PE2I BP_ND_ values at the follow-up time point in the contralateral putamen (baseline: 1.22 ± 0.07; follow-up: 1.07 ± 0.06) and the ipsilateral putamen (baseline: 1.71 ± 0.09; follow-up: 1.47 ± 0.08). Additionally, putaminal ^11^C-PE2I BP_ND_ values were significantly lower in the contralateral as compared to the ipsilateral side at both baseline and follow-up (*p* < 0.001). Further paired *t* tests showed that the mean change in ^11^C-PE2I BP_ND_ values from baseline was less prominent in the contralateral putamen (− 0.15 ± 0.16) than in the ipsilateral putamen (− 0.24 ± 0.21) (*t*_26_ = − 2.51, *p* < 0.05). The mean differences of ^11^C-PE2I BP_ND_ values between the contralateral and ipsilateral putamen were significantly smaller at follow-up (0.40 ± 0.22) than at baseline (0.49 ± 0.29) (*t*_26_ = 2.51, *p* < 0.05). In the caudate, there was a significant time × side interaction, suggesting that the effects of time on ^11^C-PE2I BP_ND_ values were different between contralateral and ipsilateral sides [*F* (1, 24) = 5.05, *p* < 0.05]. Simple effects of time (*p* < 0.001) were detected, with significantly lower ^11^C-PE2I BP_ND_ values at follow-up in both the contralateral caudate (baseline: 2.06 ± 0.15; follow-up: 1.81 ± 0.12) and ipsilateral caudate (baseline: 2.65 ± 0.17; follow-up: 2.33 ± 0.15). We also noted significant simple effects of side (*p* < 0.001), in which caudate ^11^C-PE2I BP_ND_ was lower in the contralateral side at both baseline and follow-up time points. Paired *t* tests showed that the mean changes in ^11^C-PE2I BP_ND_ values from baseline were significantly smaller in the contralateral caudate (0.25 ± 0.33) than the ipsilateral caudate (0.32 ± 0.43) (*t*_26_ = − 2.25, *p* < 0.05). The mean differences of ^11^C-PE2I BP_ND_ values between the contralateral and ipsilateral caudate were significantly smaller at follow-up (0.52 ± 0.27) than at baseline (0.59 ± 0.30) (*t*_26_ = 2.25, *p* < 0.05) (Fig. 1).

### Correlations

At baseline, significant negative correlations were found between lateralised ^11^C-PE2I BP_ND_ values and corresponding MDS-UPDRS-III scores of the clinically most (caudate: *r* = − 0.35, *p* < 0.05; putamen: *r* = − 0.33, *p* < 0.05) and least affected body sides (caudate: *r* = − 0.35, *p* < 0.05; putamen: *r* = − 0.38, *p* < 0.05). At the follow-up time point, lateralised ^11^C-PE2I BP_ND_ values did not significantly correlate with corresponding motor scores.

Lateralised changes in caudate ^11^C-PE2I BP_ND_ values from baseline negatively correlated with corresponding changes in MDS-UPDRS-III scores of the clinically most (*r* = − 0.46, *p* < 0.05) and least affected body sides (*r* = − 0.39, *p* < 0.05). Changes in MDS-UPDRS-III scores of the most affected side negatively correlated with changes in ^11^C-PE2I BP_ND_ values in the corresponding putamen (*r* = − 0.62, *p* < 0.01); however, no such correlation was found for the least affected putamen.

## Discussion

In this study, we assessed striatal dopaminergic decline and motor symptom progression in moderate PD over 19 months. We found that the effect of time on striatal ^11^C-PE2I BP_ND_ values was more pronounced in the ipsilateral as compared to the contralateral side. However, our data showed no differential effect of time on MDS-UPDRS-III scores between the clinically most and least affected body sides.

Most imaging studies on PD progression have approached striatal DAT degeneration without taking into account PD-related asymmetry [24]. To our knowledge, there have been only two longitudinal imaging studies in the literature that discuss PD asymmetry with respect to striatal imaging data [7, 9]. Nandhagopal and colleagues [7] proposed that the rate of dopaminergic decline is faster in the ipsilateral than in the contralateral striatum, especially in early and moderate stages of PD. However, the integrity of dopaminergic terminals was evaluated using ^11^C-methylphenidate, which may yield biased values depending on the severity of dopaminergic denervation [25]. Simuni and colleagues [9] noted that in the early stages of PD, changes in DAT binding in the ipsilateral putamen were more pronounced as compared to reductions seen in the contralateral putamen after a 5-year follow-up period. Nevertheless, in the latter report, the authors showed data only for the contralateral putamen, excluding useful information from the other striatal ROIs [9].

With respect to motor symptoms, a number of studies evaluating PD patients over a wide range of disease durations demonstrate retention of asymmetry over many years [10, 11]. However, two interesting studies [26, 27] using multiple regression analyses and focussing on the earlier stages of PD showed that shorter disease duration and younger age at onset correlate with higher motor symptom asymmetry. These results suggest that the progression of motor severity might not be similar across sides at the initial stages of the disease.

Our data demonstrate that the asymmetry of motor symptoms remains over 19 months in moderate PD. In contrast, a significant time-side interaction for ^11^C-PE2I demonstrates that striatal DAT asymmetry becomes less prominent over the same period. That we found significant inverse relationships between striatal DAT density and motor severity at baseline, and negative correlations between changes in ^11^C-PE2I BP_ND_ values and corresponding changes in motor scores, support the validity of this biomarker for tracking progression in moderate-stage PD. The mismatch of asymmetry trends between clinical and PET imaging data, and the failure to find a significant correlation between DAT density and corresponding motor scores at the follow-up time point, however, suggests differential patterns of progression for each aspect of the disease. Previous work describes three distinct phases of motor progression [28–30]; there is a significant improvement in motor scores attributed to treatment effects during the first two years following diagnosis, then motor scores remain relatively stable for a number of years from early to moderate PD, followed by a significant worsening of motor function in the moderate/advanced stages [28–30]. In contrast, the progressive decline in DAT density has been characterised as following an exponential decay function [31, 32]. Previous multi-tracer PET studies show that in the early stages of PD, dopaminergic compensatory adjustments, namely downregulation of DAT and upregulation of aromatic amino acid decarboxylase (AADC), are more prominent in the contralateral as compared to the ipsilateral striatum [31, 32]. However, as the disease progresses, these asymmetric compensatory responses are observed to decline [31]. Thus, the convergence of DAT density between the sides observed in the current report may be explained by a ‘floor effect’ limiting the continual reductions of the contralateral striatum in moderate PD. The exact mechanisms underlying the non-linear pattern in longitudinal compensatory evolution are unclear, but might be related to progressive and abnormal protein accumulation of α-synuclein [33] and/or chronic use of anti-parkinsonian medication [34]. Our results are in line with previous evidence, demonstrating that the development of each disease characteristic progresses according to different patterns. Aside from the degeneration of dopamine transporters, there are likely multiple other factors contributing to the pattern of motor progression, including functional changes within and beyond the dopaminergic system. Identification of novel factors for improved prediction of motor progression including those related to age, sex, baseline motor scores, cognitive function, clinical subtypes, imaging tracer (pre-and post-synaptic dopaminergic markers) and CSF biomarkers (α-synuclein, β-amyloid, and tau) is warranted.

We did not calculate an average rate of change for BP_ND_ values and MDS-UPDRS-III scores, as we do not assume a linear progression model of DAT decline in the course of PD [35]. We do not believe that aging can explain the current results as previous work demonstrates that age-related physiological decline occurs symmetrically [36]. The duration of the follow-up period (19 months) may be viewed as a methodological limitation. Although it would be ideal to continue with a third follow-up scan and/or include longer intervals between visits, we believe that the 19-month period was sufficient to reveal the change in BP_ND_ values and MDS-UPDRS-III scores in this cohort for the aims of the current study [12]. We believe that a larger number of participants would add validity to the robustness of our results. Nevertheless, we acknowledge the limitations that relate to feasibility issues, specific to the study population and the risks associated with exposure to additional ionising radiation.

We propose that while asymmetry in both striatal dopaminergic degeneration and motor symptoms continues to occur in moderate PD, its magnitude may change over the course of the disease.

## Data Availability

The datasets generated during and/or analyses during the current study are available from the corresponding author on reasonable request.
